# Antibacterial Activity of Chitosan–Polylactate Fabricated Plastic Film and Its Application on the Preservation of Fish Fillet

**DOI:** 10.3390/polym13050696

**Published:** 2021-02-25

**Authors:** Shun-Hsien Chang, Ying-Ju Chen, Hsiang-Jung Tseng, Hsin-I Hsiao, Huey-Jine Chai, Kuo-Chung Shang, Chorng-Liang Pan, Guo-Jane Tsai

**Affiliations:** 1 Institute of Food Safety and Risk Management, National Taiwan Ocean University, Keelung 20224, Taiwan; lewis@mail.ntou.edu.tw; 2 Department of Food Science, National Taiwan Ocean University, Keelung 20224, Taiwan; ingridswim@gmail.com (Y.-J.C.); hi.hsiao@mail.ntou.edu.tw (H.-IH.); b0037@mail.ntou.edu.tw (C.-L.P.); 3 Research and Development Department, Plastic Industry Development Center, Taichung 407, Taiwan; hunter0802@pidc.org.tw; 4 Seafood Technology Division, Fisheries Research Institute, Council of Agriculture, Keelung 20224, Taiwan; hjchai@mail.tfrin.gov.tw; 5 Department of Transportation Science, National Taiwan Ocean University, Keelung 20224, Taiwan; gordon@mail.ntou.edu.tw; 6 Center for Marine Bioenvironment and Biotechnology, National Taiwan Ocean University, Keelung 20224, Taiwan

**Keywords:** Chitosan, polylactic acid, antimicrobial activity, composite film, fish preservation

## Abstract

This research prepared chitosan–PLA plastic films by extrusion, analyzed the physical and mechanical properties and antibacterial activity of the fabricated plastic films, and used them to preserve grouper fillet. We added chitosan (220 kDa, 93% DD) in the weight ratio of 0.5–2% into the PLA to prepare the chitosan–PLA films. With the increasing chitosan dosage, both the water vapor transmission rate and moisture content of chitosan–PLA films increased. Among the three doses of chitosan (0.5%, 1%, and 2%) added to PLA, 0.5% chitosan–PLA film had the highest antibacterial activity. This plastic film had an inhibitory efficiency of over 95% against *Escherichia coli*, *Pseudomonas fluorescens,* and *Staphylococcus aureus*. The action of covering the fish fillet with 0.5% chitosan–PLA film significantly reduced several microbes’ counting (i.e., mesophiles, psychrophiles, coliforms, *Pseudomonas*, *Aeromonas*, and *Vibrio*) and total volatile basic nitrogen (TVBN) value in the grouper fillets stored at 4 °C. Thus, such action prolongs the fish fillets’ shelf life to up to at least nine days, and this 0.5% chitosan–PLA film shows promising potential for preserving refrigerated fish.

## 1. Introduction

Seafood is easily contaminated by microorganisms if handled and stored improperly. Due to improper on-site storage, microbial deterioration causes the loss of approximately 4–5 million tons of fish each year [1]. Various methods (i.e., chemical preservatives, irradiation, refrigeration, food packaging/films, vacuum, and nitrogen packaging) are used to prevent seafood deterioration, maintain its freshness, and extend its shelf life [2,3,4]. Synthetic plastic products are cheap, stable, and protective, so they have been widely used in food packaging and preservation. However, most synthetic plastic wraps are not biodegradable and will cause environmental pollution. Besides, many of these are petroleum-based, and potentially toxic monomers may be mixed into the food chain [5]. The impact of non-renewable and non-biodegradable petrochemical-based plastic packaging materials on the environment is increasing, arousing people’s interest in the use of biodegradable alternatives from renewable resources [6]. 

Polylactic acid (PLA) is an aliphatic polyester derived from the polymerization of lactic acid monomers. Due to its biodegradability and biocompatibility, PLA has been highly recognized and applied in biomedicine, agriculture, and packaging [7]. The US Food and Drug Administration (FDA) approved it as “Generally Recognized as Safe” (GRAS). Heat easily damages PLA’s mechanical properties, and PLA’s tensile strength is low. The traditional PLA modifying method is based on its formulation and combination with other flexible biopolymers, plasticizers, fibers, and nanofillers [8,9].

Chitosan is a biodegradable polymer obtained by exoskeleton chitin’s deacetylation from shrimp, crab, lobster, insect, and crustacean [10,11] and has attracted attention as a natural preservative due to its potent antimicrobial activities. Chitosan’s antimicrobial action appears to be mediated by the electrostatic forces between the chitosan’s protonated –NH_3_^+^ groups and the cell surfaces’ negative residues [12,13]. The number of protonated –NH_3_^+^ groups present in chitosan increases with the increased degree of deacetylation (DD). The minimum lethal concentrations (MLC) for high DD (DD ≥90%) chitosan against various food pathogens including *Bacillus cereus*, *Escherichia coli*, *Listeria monocytogenes*, *Staphylococcus aureus*, and *Vibro cholerae* are 50–200 ppm [14]. Besides chitosan’s DD, its molecular weight (MW) is another crucial factor determining its antimicrobial properties, although equivocal results in the correlation between the antibacterial properties and MWs of chitosan were also observed. Our previous report [15] concluded that the correlation between chitosan MW and its antibacterial properties depended on the reaction mixture’s pH value.

Due to the intense antibacterial activity, chitosan has been applied in the preservation of various foods, including fish [14,16], shrimp [14], oyster [17], cooked rice [11], sausages [10], and fruits [18]. However, due to chitosan’s astringency taste, either directly adding chitosan into food or dipping food in chitosan solution may cause food flavor change. Therefore, people use chitosan as food packaging materials to extend the shelf life of food. Arctic white shrimp coated with chitosan–guava peel extract had color, texture, and bacterial growth changes [13]. The 0.3% chitosan–ginger oil film (CH–GEO film) can extend barracuda (*Sphyraena jello*) fish fillet storage life at 2 °C up to 20 days [19]. However, chitosan film exhibits high moisture sensitivity and low mechanical properties. Most food applications must combine chitosan with a more moisture-resistant polymer while maintaining the product’s overall biodegradability.

Several studies have prepared chitosan–PLA films. Grande and Carvalho [20] prepared chitosan/poly(vinyl alcohol)/poly(lactic acid) films through solution mixing and film casting processes. Similarly, Sébastien et al. [21] prepared chitosan–PLA film by blending both chitosan and PLA solutions with polyethylene glycol and casting it on a glass dish. Although this chitosan–PLA film showed antifungal activity, the authors mentioned that, due to difficulties in producing the miscible PLA and chitosan film-forming solution, they obtained heterogeneous chitosan–PLA films with high water sensitivity. These features dramatically limited their further usage as packaging materials. Soares et al. [22] produced the biodegradable sheets by PLA and thermoplastic starch extrusion, coating them with a chitosan solution and then immersing them in a crosslinking agent, such as glutaraldehyde, to make a chitosan–PLA film [22]. However, there is no further study about this film’s antibacterial activity. Bonilla et al. [23] manufactured the chitosan–PLA films containing various amounts of chitosan by extrusion and showed its antibacterial application in a ground pork product.

In this study, we prepared chitosan–PLA composite films by extrusion casting compound machine. We measured the chitosan–PLA composite films’ antibacterial and physical properties and evaluated their preserving effects on grouper fillet.

## 2. Materials and Methods

### 2.1. Bacterial Strains and Chemicals

*Aeromonas hydrophila* BCRC 13881, *Escherichia coli* BCRC 11634, *Pseudomonas fluorescens* BCRC 11028, *Staphylococcus aureus* BCRC 10451, and *Vibrio parahaemolyticus* BCRC 12863 were purchased from the Biosources Collection and Research Center (Hsinchu, Taiwan). Acetic acid, acetonitrile, dimethyl sulfoxide (DMSO), glycerol, methanol, and sodium hydroxide (NaOH) were purchased from Fluka (Garage Gmbh, Buchs, Switzerland). Sodium bicarbonate (NaHCO_3_) was obtained from Sigma Chemical Co. (Gillingham, UK). Chitosan powder was obtained from Applied Chemical Co., Ltd. (Kaohsiung, Taiwan). Bacto agar, nutrient broth (NB), nutrient agar (NA), plate count agar (PCA), *Pseudomonas* isolation agar (PIA), starch ampicillin agar (SAI), thiosulphate-citrate -bile salts-sucrose (TCBS), tryptic soy broth (TSB), and violet red bile agar (VRBA) were supplied by Becton Dickinson (Sparks, MD, USA). Polylactic acid (PLA, manufactured by Natureworks@ (4032D), Minnetonka, MA, USA) exhibited a weight-average molecular weight (Mw) of 1.96 × 10^4^ Da, as determined by gel permeation chromatography. Poly(butyleneadipate-co-terephthalate) (PBAT, Ecoflex F blend C1200 with Mn of 24,400 g/mol) and ADR (ADR-4468, a chain extender) were purchased from BASF (Ludwigshafen, Germany). Talc powder (mean particle size: 8.8 μm, surface area: 5500 cm^2^/g) was purchased from Chu Shin Chemical Corp. (HsinChu, Taiwan)

### 2.2. Antimicrobial Films’ Preparation

The PLA and Chitosan–PLA films were manufactured by Plastics Industry Development Center (PIDC, Taichung, Taiwan, ROC). Both PBAT and PLA resins were fully dried with a honeycomb dehumidifying dryer (RD 100, Corsica Co., Taoyuan, Taiwan) at 160–180 °C to remove all traceable moisture to prevent potential degradation during melting processing. Chitosan powder (MW 220 KDa, as measured by size-exclusion high-performance liquid chromatography [15]; DD 93%, according to the colloid titration method [24]) was ground to about 200 μm, by using a grinder. PLA and PBAT were mixed in a ratio of 7:3, and then 2 phr (per hundreds of resin) of talc and 0.5 phr of ADR were added to obtain a base mixture. Chitosan powders were added to this base mixture to have 0.5%, 1%, and 2% in whole blends. The base mixture without chitosan addition was used as the PLA control. The extrusion of a casting laminating machine (SHFV-QA16010, HsinPow Machinery Co. Ltd., Tainan, Taiwan) produced the PLA film and chitosan–PLA composite film, which were finally trimmed into the film rolls with a 30 cm width and a 0.06 mm thickness for use.

### 2.3. Culture Conditions

*A. hydrophila* BCRC 13881, *E. coli* BCRC 11634, *P. fluorescens* BCRC 11028, and *S. aureus* BCRC 10451 were stored in NB containing 50% sterile glycerol at –80 °C. To prepare the bacteria cultures, the strains stored at –80 °C were inoculated into 50 mL NB and incubated at 37 °C for 24 h. All strains were sub-cultured twice in broths at 37 °C for 24 h.

### 2.4. Antimicrobial Activity of Films

Based on the method of ISO 22196, the antimicrobial activity of film was measured. The test sample films (50 mm × 50 mm) of PLA, PLA containing 0.5%, 1%, and 2% chitosan (0.5% CH–PLA, 1% CH–PLA, and 2% CH–PLA, respectively), and the covered plastic film (40 mm × 40 mm) were sterilized by UV light for 24 h before use. The test films were added with 0.4 mL of the diluted culture (2.5–10 × 10^5^ CFU/mL), covered with the plastic films, and lightly pressed to spread the culture without overflowing it. After incubation at 37 °C for 24 h, the test films were rinsed with 10 mL of SCDLP (Soybean casein digest broth with lecithin and polyoxyethylene sorbitan monooleate) broth. The control PLA film was tested in parallel with the addition of bacterial culture, covered with a plastic film, and immediately rinsed with SCDLP broth. The viable bacterial count in the washed SCDLP broth was measured by counting colonies on the PCA plate. The test was conducted in triplicate.

### 2.5. Application of the Active Films on the Preservation of Fish Fillet

Based on the method of Remya et al. [19] with some modification, locally purchased fresh grouper (*Epinephelus fuscoguttatus* x *Epinephelus lanceolatus*) were cut into small fillets (30 g each, 5 × 2 × 3 cm^3^), and the upper and lower surfaces of the fish fillets were coated with test films (7 cm × 7 cm). Non-coated (control) and coated samples were placed in Petri dishes and stored at 4 and 25 °C. At intervals, five fish samples from each condition were removed, in two of which total volatile basic nitrogen (TVBN) contents were measured using the microdiffusion method [25]. The other three samples were subjected to pH measurement and microbial analysis. Based on the method of Tsai et al. [14], decimal diluents (100 μL) were spread in duplicate on various media and incubated as follows: PCA at 30 °C and 48 h for the aerobic mesophilic count and at 7 °C and 10 dayd for the psychrotrophic count; VRBA at 37 °C and 24 h for the coliform count; SAI at 28 °C and 24 h for the *Aeromonas* count; PIA at 26 °C and 24 h for the *Pseudomonas* count; and TCBS at 26 °C and 24 h for the *Vibrio* count.

### 2.6. Films’ Physical and Mechanical Properties Determination

#### 2.6.1. Water Vapor Permeability (WVP)

Based on Wang et al.’s method [26], a test tube (depth 8 cm × diameter 4 cm) containing anhydrous silica gel was sealed with the test film, and placed in a controlled environment (20 ± 2 °C and 50 ± 5% RH). The water vapor transmission rate (g mm/m^2^ day kPa) was calculated from the test tube’s increased weight over time at steady state of transfer. The test was conducted in triplicate.

#### 2.6.2. Moisture Content

A precisely weighed film sample (50 mg) was dried to constant weight at 105 °C [26]. The film’s moisture content was expressed as a percentage of film dry weight reduction after drying, as compared to the initial film weight. The test was conducted in triplicate.

#### 2.6.3. Solubility

Film solubility was measured from immersion assay in distilled water at 25 °C for 24 h [26]. Film solubility was expressed as a percentage of film dry weight reduction after immersion, as compared to the initial film dry weight. The test was conducted in triplicate.

#### 2.6.4. Mechanical Properties

Film tensile test was performed according to the standard method of ASTM D882 [27]. The films’ tensile strength and elongation at break were calculated from the stress-strain curves. Films’ tear strength was measured according to ASTM D1938.

### 2.7. Statistical Analysis

In this study, all data were expressed as mean values with standard deviation (mean ± SD). Statistical analysis was carried out using SPSS 16.0 software (SPSS, Chicago, IL). All data were analyzed statistically using a repeated measure and one-way analysis of variance (ANOVA), and multiple comparisons between treatment means were completed by Duncan’s tests. All experiments were carried out in triplicate, and the statistical significance level of *p* < 0.05 was considered.

## 3. Results and Discussion

### 3.1. Mechanical and Physical Properties of Films

**Figure 1:**
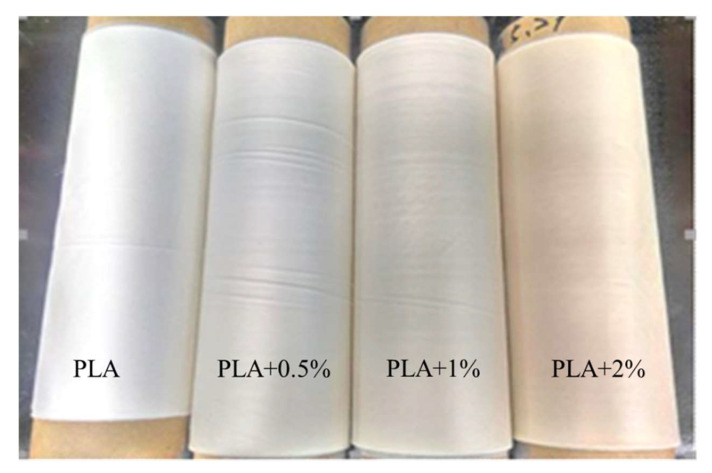
Appearance of the film rolls of PLA and PLA containing 0.5%, 1%, and 2% of chitosan.

The visual appearance of pure PLA films and chitosan–PLA composites films are shown in Figure 1. The PLA film is more transparent than the chitosan–PLA composite films, and this is consistent with the observations previously reported in [23]. The chitosan–PLA films are yellowish, which may be due to chitosan’s partial miscibility affecting the continuous matrix’s color. The film’s tensile strength, the elongation at break, and the tearing strength are shown in Table 1.

The test groups’ tensile strengths of machine direction (MD) and transverse direction (TD) were 448–155 and 271–79 kgf/cm^2^, respectively. The chitosan concentration’s increase in PLA decreased the tensile strength, and. therefore, 0.5% chitosan–PLA film had the best tensile strength among all chitosan–PLA composite films. The addition of chitosan particle may result in irregularities and discontinuities in the oriented PLA matrix [23], which caused the decrease of chitosan–PLA film’s tensile strength. However, in this study, when the chitosan concentration was 0.5%, the PLA’s MD elongation at break increased, although its value slightly decreased when the chitosan concentration was further increased. Besides, adding chitosan to PLA significantly increased both the MD and TD tearing strength (gf). Although we are currently unable to provide the correct reason, the helical configuration [28] and the size of chitosan molecule may help increase the elongation and tear strength of PLA film containing appropriate amount of chitosan.

Table 2 shows both PLA and chitosan–PLA films’ water vapor transmission rate, moisture content, and solubility. With the increasing concentration of chitosan, the PLA films’ water vapor transmission rate and moisture content increase, with 2% chitosan–PLA film being the best one. This result may be due to chitosan’s hydrophilic properties [26]. In contrast, due to the PLA’s hydrophobicity, Suyatma et al. [29] proved that PLA addition reduced the chitosan–based films’ water vapor transmission rate. The solubility of test films was quite low and there was no significant difference among them.

### 3.2. Antimicrobial Activity of Films

**Figure 2:**
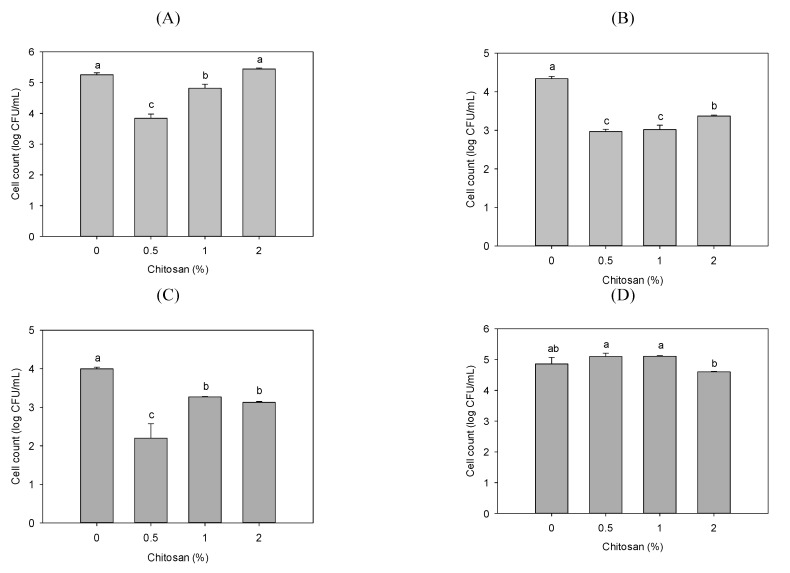
Antibacterial activity of various films of PLA, and PLA containing 0.5%, 1%, and 2% of chitosan against: (**A**) *Escherichia coli;* (**B**) *Staphylococcus aureus*; (**C**) *Pseudomonas fluorescens*; and (**D**) *Vibrio parahaemolyticus*. Data are presented as mean ± SD (*n* = 3). Bars with different letters on top are significantly different (*p* < 0.05).

Figure 2 shows the PLA and 0.5%, 1.0%, and 2.0% chitosan–PLA films’ antimicrobial activities against *E. coli*, *S. aureus*, *P. fluorescens*, and *V. parahaemolyticus*. Except for *V. parahaemolyticus*, 0.5% chitosan–PLA film showed the highest activity. This plastic film’s inhibition efficiency against *E. coli*, *P. fluorescens*, and *S. aureus* was over 95%. In addition, among the four target bacteria, *P. fluorescens* seems to be more susceptible to chitosan–PLA films. Except for *V. parahaemolyticus*, the activity of higher chitosan concentrations (1.0% and 2.0%) in PLA was significantly lower than that of lower chitosan concentrations (0.5%). Several reports have shown that the presence of more -NH_3_^+^ residues in chitosan is conducive to binding to bacterial cells, resulting in bacterial structural instability [12,15,30,31,32]. The pKa value for chitosan is approximately 6.3–6.5, dependent on chitosan’s MW [33]. We speculate that, when the diluted bacterial culture is added to the film surface, some of the amine residues protruding from the film surface are protonated, thereby having antibacterial activity. However, when the content of chitosan in the film increases, it may cause the film structure to become tighter, hinder the exposure of amine groups, or generate electrostatic attraction within or between chitosan molecules [28], thereby reducing its antibacterial activity. In addition, our previous study demonstrated that the chitosan’s antimicrobial activity varies considerably with the DD, MW, and reaction pH [15]. Since we only used one chitosan sample with 93% DD and 220 kDa in this study, the comprehensive effects of chitosan DD, MW, and concentration on the chitosan–PLA film’s structure and antibacterial activity merit future investigation.

In short, 0.5% chitosan–PLA film has better tensile strength, elongation at break, and antibacterial activity. Therefore, we selected it for the fish fillet preservation test.

### 3.3. Chitosan–PLA Film’s Application for Preservation Fish Fillet

**Figure 3:**
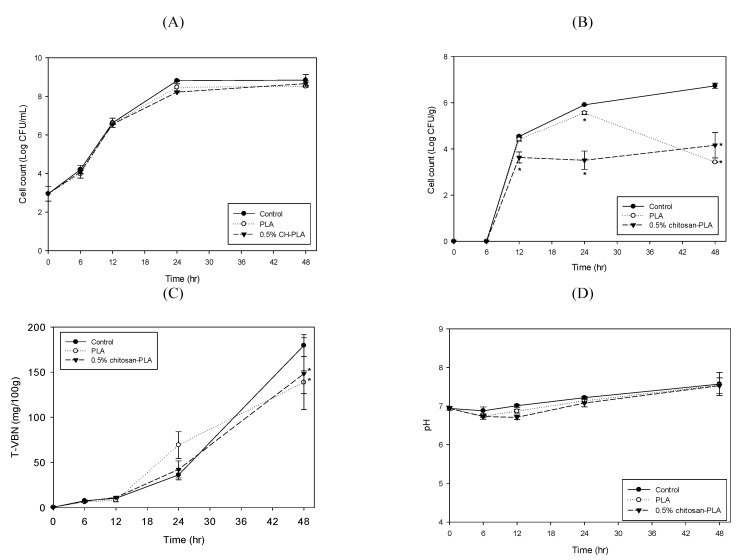
Changes in mesophilic count (**A**); psychrotrophic count (**B**); total volatile basic nitrogen content (TVBN) (**C**); and pH value (**D**) of fish fillet (*Epinephelus fuscoguttatus x Epinephelus lanceolatus*) uncovered (control) or covered with PLA or 0.5% CH–PLA films during storage at 25 °C. Data are presented as mean ± SD (*n* = 3). * significantly different compared to control (*p* < 0.05).

Due to the fresh fish products’ high perishable characteristics, chitosan has been used to extend the salmon fish fillet’s shelf life [14]. Our study evaluated the PLA and the 0.5% chitosan–PLA films application’s preservation effects on grouper fish fillets stored at 25 °C and 4 °C. Figure 3 shows the differences among fish fillets uncovered with film (control) or covered with PLA or 0.5% chitosan–PLA (0.5% CH–PLA) films during storage at 25 °C. Specifically, we measured the samples’ changes in the mesophilic count, psychrotrophic count, TVBN content, and pH value. The mesophilic counts of the control, PLA, and chitosan–PLA groups were similar, although the mesophilic counts of fish fillets covered with a 0.5% chitosan–PLA film after 24 h incubation were slightly lower than other fish fillets (Figure 3A). After 24 h of incubation, the psychrotrophic count in the 0.5% chitosan–PLA group dropped to 3.51 Log CFU/g, which was significantly lower than the control (5.91 Log CFU/g) and PLA (5.56 Log CFU/g) groups. After 48 h incubation, the psychrotrophic count of the 0.5% chitosan–PLA group remained at about 4.0 Log CFU/g, which was significantly lower than that of the control (Figure 3B). The TVBN is a quantitative parameter to determine the content of ammonium and type I, II, and III amines in fish. An increase in TVBN indicates fish and bacterial enzyme actions increase, leading to fish spoilage [34]. Figure 3C shows the TVBN value changes of uncovered control fish fillets and fish samples covered with PLA and 0.5% chitosan–PLA film. The TVBN of the 0.5% chitosan–PLA group was significantly lower than that of the control and PLA group after 48 h of incubation (Figure 3C). There was no difference in pH between the test groups (Figure 3D).

**Figure 4:**
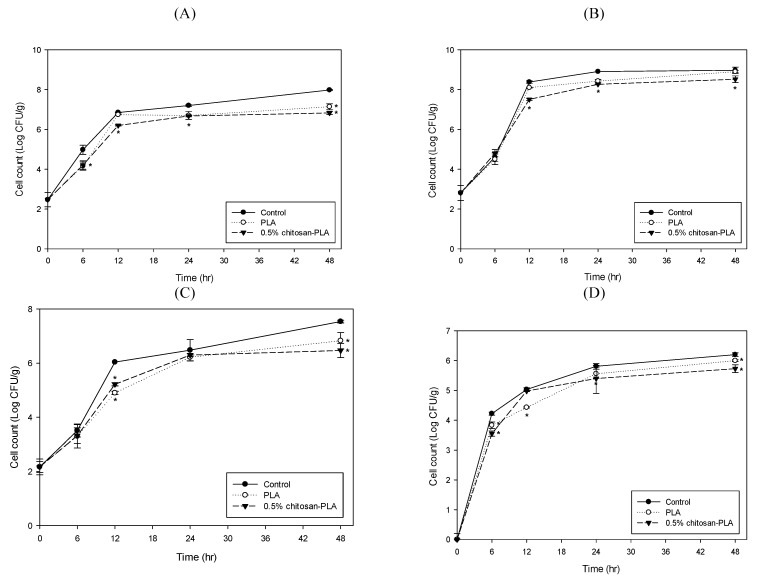
Changes in cell counts of coliforms (**A**); *Aeromonas* (**B**); *Pseudomonas* (**C**); and *Vibrio* (**D**) groups in fish fillet (*Epinephelus fuscoguttatus* x *Epinephelus lanceolatus*) uncovered (control) or covered with PLA or 0.5% CH–PLA film during storage at 25 °C. Data are presented as mean ± SD (*n* = 3). * significantly different compared to control (*p* < 0.05).

Figure 4 shows the cell count changes of various microbiomes in fish fillet covered with PLA or 0.5% chitosan–PLA film during the 48 h of storage at 25 °C. After 12 and 48 h of incubation, the coliform counts (Figure 4A) and *Aeromonas* counts (Figure 4B) for the 0.5% chitosan–PLA film group were significantly lower than those of the uncovered control group. After 12 h of incubation, the *Pseudomonas* counts for PLA and 0.5% chitosan–PLA film groups were 4.9 and 5.2 Log CFU/g, respectively, which were significantly lower than that of the control group (over 6 Log CFU/g) (Figure 4C). The sample groups’ *Vibrio* counts were comparably lower than the control group (Figure 4D).

**Figure 5:**
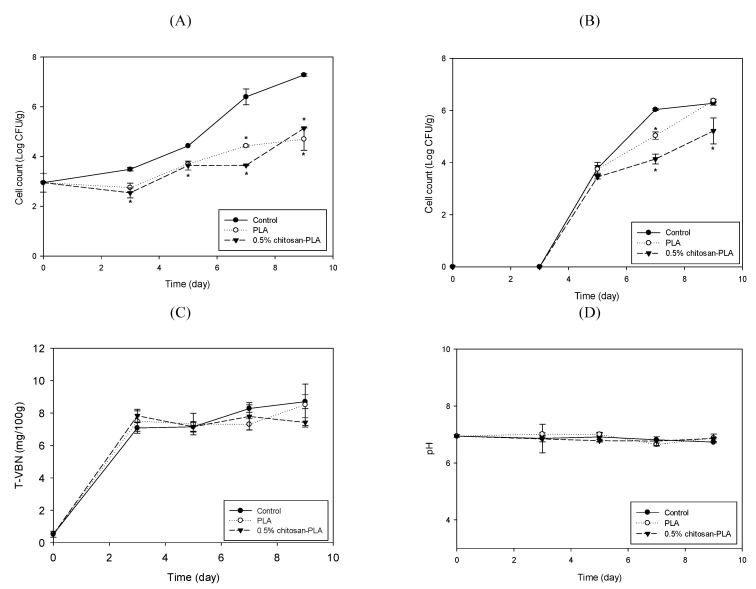
Changes in mesophilic count (**A**); psychrotrophic count (**B**); total volatile basic nitrogen content (**C**); and pH value (**D**) of fish fillet (*Epinephelus fuscoguttatus x Epinephelus lanceolatus*) uncovered (control) or covered with PLA or 0.5% CH–PLA films during storage at 4 °C. Data are presented as mean ± SD (*n* = 3). * significantly different compared to control (*p* < 0.05).

Figure 5 shows the changes in the mesophilic count, psychrotrophic count, TVBN, and pH value of fish fillet uncovered with film (control) or covered with PLA or 0.5% chitosan–PLA films (0.5%CH–PLA) during storage at 4 °C. At least three days after storage, covering fish fillets with PLA or 0.5% chitosan–PLA film effectively inhibited the increase in mesophilic bacteria count. After seven days of storage, the control’s mesophilic count exceeded 6 Log CFU/g (control limit), while the count for the 0.5% chitosan–PLA group was 3.16 Log CFU/g, which was significantly lower than that of control and PLA groups. The PLA and 0.5% chitosan–PLA groups’ counts after nine days were still below the control limit (Figure 5A). In all groups, there was no psychrotrophic growth during the first three days of storage. After nine days of storage, the 0.5% chitosan–PLA group’s psychrotrophic counts were significantly lower than those of the control and PLA groups (Figure 5B). After nine days of storage at 4 °C, the TVBN content of all tested fish samples was below 10 mg/100 g, which is far below the control limit for raw fish (25 mg/100 g). By the ninth day, the TVBN content of fish fillets covered with a 0.5% chitosan–PLA film was 7.42 mg/100 g, which was significantly lower than the control and PLA groups (Figure 5C). There was no significant difference in all groups’ pH values after nine storage days (Figure 5D).

**Figure 6:**
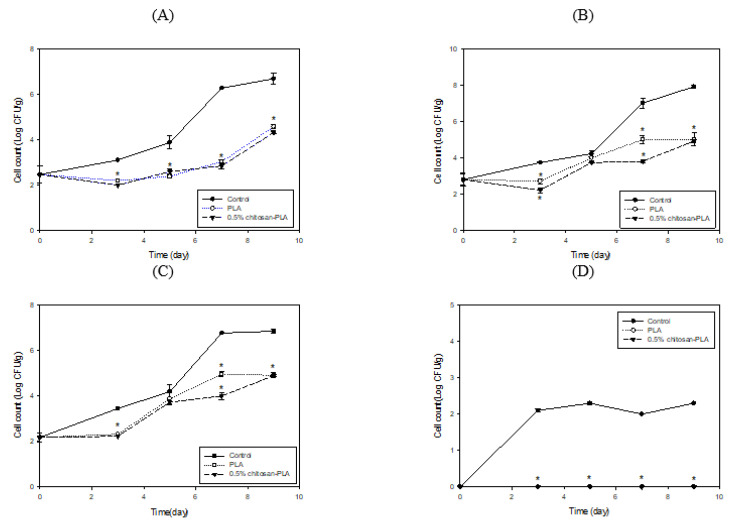
Changes in cell counts of coliforms (**A**); *Aeromonas* (**B**); *Pseudomonas* (**C**); and *Vibrio* (**D**) groups of fish fillet (*Epinephelus fuscoguttatus x Epinephelus lanceolatus*) uncovered (control) or covered with PLA or 0.5% CH–PLA film during storage at 4 °C. Data are presented as mean ± SD (*n* = 3). * significantly different compared to control (*p* < 0.05).

When stored at 4 °C for nine days, the PLA and 0.5% chitosan–PLA films effectively retarded the increase in the counts of coliform, *Aeromonas,* and *Pseudomonas* in the fish fillets. Especially when stored for seven days, these bacterial counts of fish fillets covered with 0.5% chitosan–PLA film were all lower than 4Log CFU/g (Figure 6A–C). The *Vibrio* counts in PLA and 0.5% chitosan–PLA film covered fish fillets during storage at 4 °C were not detectable, while the *Vibrio* count in control was about 2 Log CFU/g (Figure 6D) during storage at 4 °C.

Chitosan has antibacterial activity and non-toxic properties and has been demonstrated to be an ideal antibacterial packaging material. Remya et al. [19] prepared chitosan–ginger essential oil film (CH–GEO film) and demonstrated that covering it with CH–GEO film effectively reduced the fish fillet’s (*Sphyraena jello*) TVBN content and the mesophilic count. This process effectively prolonged the fish fillets’ shelf life at 2 °C up to 20 days. Chitosan–polyethylene film was shown to have good antibacterial activity against *E. coli*, *Listeria monocytogenes,* and *Salmonella enteritidis*, thereby effectively extending the refrigeration beef’s shelf life [25]. Fathima et al. [35] used ethylene glycol and vinyl alcohol as crosslinkers and plasticizers to prepare nano-chitosan–PLA film. They further demonstrated that covering this film could effectively prolong Indian white prawn’s (*Fenneropenaeus indicus*) shelf life. In this study, 0.5% chitosan–PLA film led to significant reduction of microbes in fish fillets stored at 4 and 25 °C; especially when compared with 25 °C, the reduction in fish fillets observed at 4 °C was greater. In addition, the PLA film also showed a slight decrease in microbial numbers in the fish fillet, which may be related to the oxygen impermeability of the PLA film [23]. In summary, the overall effects of the chitosan in the PLA film, the low temperature environment, and the low oxygen permeability of the film led to the chitosan–PLA film greatly reducing the bacteria in the fish fillet and extending its shelf life at 4 °C.

## 4. Conclusions

Although chitosan addition could reduce the PLA film’s tensile strength, the PLA film containing 0.5% chitosan still maintained acceptable tensile strength, had increased elongation at break, and strong antimicrobial activity. The 0.5% chitosan–PLA film covering could effectively reduce the mesophiles, coliforms, and spoilage bacteria counts, as well as the TVBN content in fish fillets stored at 4 °C. Given the consumer’s demand for natural preservatives and the need to replace petroleum-based plastic packaging with biodegradable and eco-friendly alternatives, the 0.5% chitosan–PLA film shows promising potential for preserving fish and other food.

## Figures and Tables

**Table 1:** Tensile strength, elongation at break, and tearing strength of PLA and Chitosan–PLA films.

Composition	Tensile Strength (kgf/cm^2^)		Elongation at Break (%)		Tearing Strength (gf)	
	MD	TD	MD	TD	MD	TD
PLA	448 ± 19 ^a^	271 ± 18 ^a^	318 ± 16 ^b^	498 ± 41 ^a^	54 ± 7 ^c^	220 ± 14 ^c^
0.5% CH–PLA	261 ± 23 ^b^	139 ± 8 ^b^	376 ± 37 ^a^	416 ± 39 ^a^	81 ± 7 ^b^	282 ± 14 ^a^
1% CH–PLA	216 ± 3 ^c^	133 ± 12 ^b^	320 ± 7 ^b^	414 ± 67 ^a^	97 ± 12 ^a^	252 ± 13 ^b^
2% CH–PLA	155 ± 8 ^d^	79 ± 6 ^c^	255 ± 11 ^c^	271 ± 32 ^b^	84 ±8 ^b^	226 ± 24^c^

MD, machine direction; TD, transverse direction; 0.5% CH–PLA, 1% CH–PLA, and 2% CH–PLA, PLA containing 0.5%, 1%, and 2% chitosan, respectively. Data are presented as mean ± SD (*n* = 3). Different superscript in the same column indicates significant difference (*p* < 0.05).

**Table 2:** Water vapor transmission rate, moisture content, and film solubility of PLA and Chitosan–PLA films.

Composition	Water Vapor Transmission Rate (g mm/m^2^ day kPa)	Moisture Content (%)	Film Solubility (%)
PLA	0.53 ± 0.00 ^d^	0.26 ± 0.37 ^b^	0.82 ± 0.68 ^a^
0.5% CH–PLA	0.71 ± 0.03 ^c^	1.68 ± 0.32 ^a^	1.73 ± 1.01 ^a^
1% CH–PLA	0.96 ± 0.02 ^b^	2.09 ± 1.81 ^a^	0.49 ± 1.33 ^a^
2% CH–PLA	1.25 ± 0.36 ^a^	2.62 ± 0.35 ^a^	0.23 ± 0.97 ^a^

0.5% CH–PLA, 1% CH–PLA, and 2% CH–PLA: PLA containing 0.5%, 1%, 2% chitosan, respectively. Data are presented as mean ± SD (*n* = 3). Different superscript in the same column indicates significant difference (*p* < 0.05).

## Data Availability

The data used to support the findings of this study are available from the corresponding author upon request.
